# Telemedicine in allergy/immunology in the era of COVID-19: a Canadian perspective

**DOI:** 10.1186/s13223-022-00657-3

**Published:** 2022-02-21

**Authors:** Sarah Edgerley, Rongbo Zhu, Ariba Quidwai, Harold Kim, Samira Jeimy

**Affiliations:** 1 Division of Clinical Immunology and Allergy, Department of Medicine, Western University, London, ON Canada; 2 Schulich School of Medicine and Dentistry, Western University, London, ON Canada; 3 Division of Clinical Immunology and Allergy, Department of Medicine, McMaster University, Hamilton, ON Canada

**Keywords:** Telemedicine, Telehealth, Allergy, Immunology, COVID-19, Virtual care

## Abstract

**Background:**

In the era of COVID-19, utilization of telemedicine has dramatically increased. In addition to reduced travel times, patient expenses, and work or school days missed, telemedicine allows clinicians to provide continued care while minimizing face-to-face interactions, maintaining social distancing, and limiting potential COVID-19 exposures. Clinical Immunology and Allergy (CIA), like many specialties, has adapted to incorporate telemedicine into practice. Previous studies have demonstrated similar patient satisfaction between virtual and in-person visits. However, evidence from fully publicly funded health care systems such as Canada has been limited.

**Methods:**

We performed a quality improvement (QI) initiative to assess the feasibility of telemedicine. Between 1 March and 30 September 2020, patient encounters of two academic allergists at a single institution in London, Ontario, Canada were analyzed. Assessments were categorized into in-person or telemedicine appointments. A random sample of patients assessed virtually completed a voluntary patient satisfaction survey. Qualitative analysis was performed on survey comments.

**Results:**

In total 3342 patients were seen. The majority were adults (n = 2162, or 64.7%) and female (n = 1872, or 56%). 1543 (46.2%) assessments were virtual and 1799 (53.8%) assessments were in-person. 67 of 100 random patient surveys sent to those in the virtual assessment group were completed. 89.6% (n = 60) agreed or strongly agreed when asked if they were satisfied with their telemedicine visit. 64.2% (n = 43) felt they received the same level of care compared to in-person assessments and 91% (n = 61) stated they would attend another virtual appointment. 95.4% (n = 62) of patients reported saving time with virtual assessment, the majority (n = 42, 62.7%) estimating between 1–4 h saved. Reported shortcomings included technical difficulties, “feeling rushed”, and missing in-person interactions.

**Conclusions:**

Our quality improvement initiative demonstrated high patient satisfaction and time savings with virtual assessment in a publicly funded health care system. Studies suggest that CIA may be uniquely situated to benefit from permanent integration of virtual care into regular practice for both new and follow-up appointments. We anticipate continued increased utilization of telemedicine, signifying a lasting beneficial change in the delivery of healthcare.

## Background

In response to the COVID-19 pandemic, unprecedented changes and progress have occurred in the distribution of healthcare services. One area at the forefront of advancement is virtual delivery of care through telemedicine [1, 2]. Previously prioritized for patients living in remote areas with limited access to in-person medical care, telemedicine has emerged as a viable option for even those living in urban settings [3]. Telemedicine reduces travel times, patient expenses, and work or school days missed [4, 5]. In context of the global pandemic, it allows clinicians to provide continued care while minimizing face-to-face interactions, maintaining social distancing, and limiting potential COVID-19 exposures [2].

While the use of virtual medicine in Clinical Immunology and Allergy (CIA) was increasing prior to the COVID-19 pandemic, CIA, like many specialties, has recently adapted to further incorporate telemedicine into practice [1, 2, 6]. CIA specific studies from multiple countries have shown similar patient satisfaction between virtual and in-person visits [1, 6–9]. However, for telemedicine, evidence from fully publicly funded health care systems such as Canada has been limited. A recent Canadian study by Shiff et al. did demonstrate patient satisfaction to telephone appointments as an alternative at a urology clinic [10]. However, to date, little is known regarding CIA telemedicine and patient satisfaction in Canada.

## Methods

We performed a quality improvement (QI) initiative to assess the feasibility of telemedicine. Between 1 March and 30 September 2020 inclusively, we analyzed patient encounters of two academic allergists at a single institution in London, Ontario, Canada. Assessments were categorized into in-person or telemedicine appointments. All telemedicine appointments were synchronous via telephone or video, scheduled at 15–20 min intervals. Patient demographics including gender, age (adult [≥ 18 years old] or pediatric [< 18 years old]), and primary diagnosis based on Ontario Health Insurance Plan (OHIP) Diagnostic codes (determined by the physician as the reason for consultation) were documented [11]. To determine patient perception of telemedicine, a random sample of initial consultations assessed virtually completed a voluntary patient satisfaction survey. For pediatric patients, these surveys were sent to the caregiver. Qualitative analysis was performed on submitted comments by 2 of the authors and confirmed by the rest of the authors of this manuscript.

## Results

Between 1 March to 30 September 2020, 3342 patients were assessed at the academic centre. The majority of patients were adults (n = 2162, or 64.7%) and female (n = 1872, or 56%). 1543 (46.2%) assessments were virtual and 1799 (53.8%) assessments were in-person. The most common primary diagnoses were rhinitis (n = 788, or 23.6%), anaphylaxis (n = 637, or 19%), hives (n = 531, or 15.9%), adverse effects of drugs (n = 471, or 14.1%), and asthma (n = 464, or 13.9%) (Table 1).

**Table 1:** Patient demographics

	All assessments	Virtual assessments	In-person assessments
Number of patients, n (%)	3342	1543 (46.2%)	1799 (53.8%)
Sex, n (%)			
Female	1872 (56%)	890 (57.7%)	982 (54.6%)
Male		653 (42.3%)	817 (45.4%)
Age, n (%)			
<18 years		458 (29.7%)	722 (40.1%)
≥18 years		1085 (70.3)	1077 (59.9%)
Primary diagnosis, n (%)			
Rhinitis	788 (23.6%)	329 (21.3%)	459 (25.5%)
Anaphylaxis	637 (19%)	230 (14.9%)	407 (22.6%)
Hives	531 (15.9%)	329 (21.3%)	202 (11.2%)
Adverse effects of drugs	471 (14.1%)	211 (13.7%)	260 (14.5%)
Asthma	464 (13.9%)	213 (13.8%)	251 (14%)
Bites, venomous	158 (4.7%)	40 (2.6%)	118 (6.6%)
Respiratory system, other	108 (3.2%)	87 (5.6%)	21 (1.2%)
Atopic dermatitis	54 (1.6%)	30 (1.9%)	24 (1.3%)
Seborrheic dermatitis	51 (1.5%)	26 (1.9%)	25 (1.4%)
Immunity disorder	51 (1.5%)	31 (2%)	20 (1.1%)
Other	29 (0.9%)	17 (1.1%)	12 (0.7%)

**Fig. 1:**
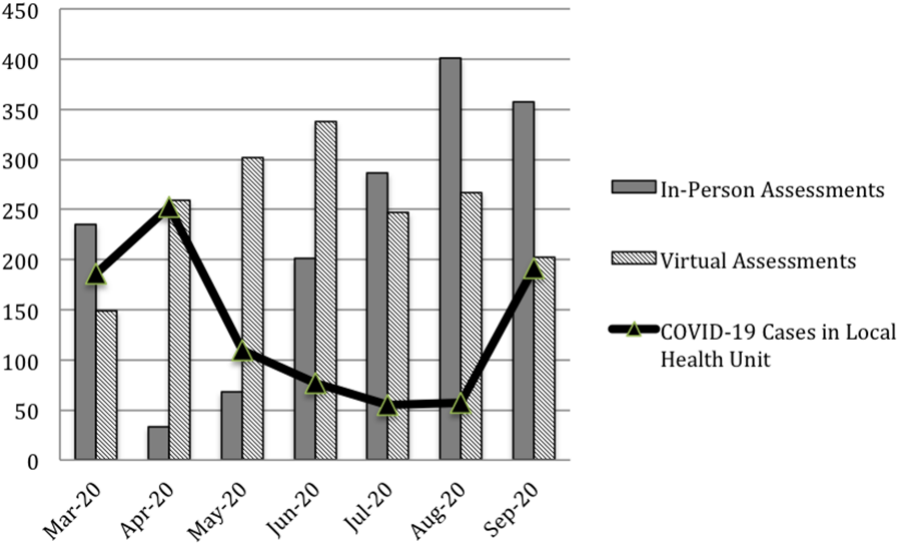
Clinical assessment type and local COVID-19 case numbers by month

Publicly available data from the local health unit was analyzed and the number of COVID-19 cases in the region was documented [12]. Overall case number trends in Ontario mirrored those of the local public health unit [13]. Figure. 1 displays by month the number of COVID-19 cases in the region and the number of assessments (in-person or virtual) completed by the clinic.

Out of 100 random patient surveys sent in the virtual assessment group (Table 2), 67 patients completed the survey. 60 patients (89.6%) agreed or strongly agreed when asked if they were satisfied with their telemedicine visit. 43 (64.2%) patients felt they received the same level of care compared to in-person visits. 55 (82.1%) believed telemedicine should be offered to all patients and 61 (91.0%) stated they would attend another telemedicine visit. 62 (95.4%) patients reported saving time with telemedicine assessment, the majority estimating between 1–4 h saved (n = 42, or 62.7%). Noted benefits of virtual clinics included shortened wait times and improved access to care, while shortcomings included technical difficulties, “feeling rushed”, and missing in-person interactions.

**Table 2:** Patient survey results. Total number of respondents/N = 67

I was satisfied with my telemedicine clinic visit	
Strongly agree	29 (43.3%)
Agree	31 (46.3%)
Neither agree or disagree	6 (9.0%)
Disagree	0 (0%)
Strongly disagree	1 (1.5 %)
I felt I received the same level of care from telemedicine as I would if I attended clinic in person	
Strongly agree	14 (20.9%)
Agree	29 (43.3%)
Neither agree or disagree	12 (17.9%)
Disagree	10 (14.9%)
Strongly disagree	2 (3.0%)
I saved time out of my day attending telemedicine compared to in person clinic	
Strongly agree	62 (92.5%)
Agree	5 (7.5%)
How much time did you save?	
<1 hour	14 (20.9%)
1–2 hours	25 (37.3%)
2–4 hours	17 (25.4%)
>4 hours	6 (9.0%)
I did not save any time	5 (7.5%)
Do you think telemedicine should be offered to all patients?	
Yes	55 (82.1%)
No	12 (17.9%)
Would you attend another telemedicine clinic?	
Yes	61 (91.0%)
No	6 (9.0%)

## Discussion

This is the first Canadian analysis of the implementation and continuation of telemedicine in a CIA practice during the COVID-19 pandemic. We note high levels of patient satisfaction after virtual assessment. 89.6% of survey respondents were satisfied with their telemedicine assessment, 82.1% believing virtual appointments should be offered to all patients, and 91% stating they would attend another virtual clinic. These high satisfaction rates are similar to results from previous virtual CIA studies from the US and Europe [1, 5, 7, 8]. Lanier et al. noted that 77% of virtually assessed patients would strongly recommend telemedicine to others, with 46% indicating a preference for telemedicine over in-person visits even after the pandemic ends [8]. Thomas et al. determined that 85% of CIA patients assessed virtually thought that the overall experience was “good/very good”, and 75% felt they were able to get as much out of a virtual assessment as an in-person appointment [1]. Mustafa et al. found that 97% of patients surveyed were satisfied with their virtual CIA appointment, and 77.4% felt that it was as satisfactory as an in-person assessment [7]. 98.8% of surveyed CIA patients in Waibel et al.’s study recommended tele-allergy and reported high satisfaction levels [5].

Qualitative data from our voluntary patient surveys highlighted perceived benefits including improved access to care and shortened wait times. Previous research has also identified significant time and cost benefits of virtual medicine in Canada across different medical specialties. Appireddy et al. reported an estimated 1.33 h, 30.1 km, and $52.83 in patient savings per virtual stroke prevention clinic appointment in Kingston, Ontario, Canada [14]. Berlin et al. Toronto, Ontario, Canada study estimated between 35.2–43 km and $136.50–$142.90 in patient savings per virtual cancer care appointment during the COVID-19 pandemic [15]. Habashi et al. found that patients living in rural Ontario, Canada saved an average of $767.18 per virtual gastroenterology assessment [16]. Lee et al. determined an average potential travel time of 6.6 h saved per virtual CIA appointment in Toronto, Ontario, Canada [4]. Although there are no Canadian CIA cost analysis studies at the time of this manuscript, a US study by Waibel et al. estimated potential patient savings of $485 USD in travel expenses, 438 driving miles, and 2.3 days of work or school per virtual CIA appointment [5]. 95.4% of our survey respondents reported time-savings with virtual assessment, the majority estimating between 1–4 h saved (n = 42, or 62.7%) with a median of 2–3 h saved per appointment. This equates to about 3528–7056 patient hours saved based on the virtual patients seen in our initiative.

However, 17.9% (n = 12) of our respondents did not feel like they received the same level of care as in-person visits and 1.5% (n = 1) were strongly dissatisfied after their telemedicine appointment. 9% (n = 6) indicated they would not attend another virtual appointment. Qualitative analysis identified technical difficulties, the feeling of being rushed, and missing in-person interactions as common themes for negative experiences with virtual assessments. These sentiments are similar to Thomas et al.’s study findings which noted negative experiences including feeling that the virtual assessment was “impersonal” compared to a face-to-face visits, low audio quality, and the need for a follow up in-person assessment for allergy testing [1]. Mustafa et al. found that the most commonly reported reason patients preferred face-to-face assessments was the desire for more personal interactions [7]. Our virtual appointments were booked at 15–20 min intervals. In the future, providing longer appointment times for virtual appointments may help reduce the feeling of being rushed. However, Thomas et al. retrospectively reviewed 537 virtual CIA appointments with an allocated appointment time of 20 min [1]. 1% of survey respondents after these assessments felt that the appointment duration was “about right” [1]. There are likely additional factors other than appointment duration that should be addressed to improve the virtual interaction. Moving forward, as telemedicine continues to be integrated into medical care, there should be a focus on minimizing technical disruptions and the perception of reduced interactions or compromised doctor-patient relationships [3].

In our clinic, virtual assessments continued at high levels even after temporary reductions in local COVID-19 case numbers and easing of restrictions (Fig. 1). Perhaps this indicates patient and/or provider satisfaction or preference for virtual visits. This data suggests that virtual assessments may continue at high levels post-pandemic even as overall case numbers decline and restrictions lift.

Our QI initiative does have a few limitations. We were not able to send surveys to all virtually-assessed patients and given the anonymized and randomization of the survey results, we are unable to know whether the respondents are an adequate representation of the general population. However, we believe the sample from our randomly sent surveys and comments are representative of overall perception of virtual care as our results are in keeping with similar studies [1, 5–9, 17]. We were not able to record other medical comorbidities of the patients given the methodology of our QI initiative. This is in keeping with previous similar CIA virtual care patient satisfaction survey studies [1, 5–8, 17]. While our patient surveys were only sent to new consultation patients, Mustafa et al. noted similar high rates of patient satisfaction between new and follow-up patients in their study [7].

Our study did not assess patient outcomes or completeness of virtual encounters, but other studies have demonstrated that the virtual telemedicine modality is effective for both new and follow-up appointments in CIA. Thomas et al. determined that over 42% of new virtual allergy/immunology consults could be discharged from service after initial telemedicine assessment [1]. Waibel et al. found that over 75% of virtual CIA consultations did not require in-person follow up [5]. This indicates that a large proportion of new assessments can be completed without in-person visits or testing, likely because certain diagnoses are determined primarily through clinical history and review of medical records [3]. Studies have also demonstrated that CIA telemedicine follow-up appointments are effective, with fewer than 10% requiring in-person follow-up visits [1, 5].

Despite the many advantages of virtual care, certain consultations will still require in-person assessment to help facilitate procedures such as skin testing and oral food or drug challenges. However, even for patients who do require in-person reassessment, initial virtual evaluation is highly beneficial and can lead to improved productivity and efficiency. Virtual assessments afford clinicians the opportunity to order relevant investigations, start medical therapy and evidence based management, and provide patient education [1–3, 18]. By the time the patient is seen for in-person follow-up, all relevant investigations would be available and response to management could be assessed [6]. Any relevant drug/medication preparations not typically stocked in clinic could also be ordered by this time for skin prick testing, intradermal testing, or drug challenges [1]. Certain patient presentations including chronic urticaria, non-IgE-mediated food reactions, atopic dermatitis, historical adverse drug reactions, asthma, allergic rhinitis, and immunologic conditions are thought to be particularly well suited for virtual assessment [2, 3, 6, 18]. Future studies are needed to help address cost benefit analysis of telemedicine in Canada as well as its impact on wait times especially in a fully publicly funded health care system.

## Conclusions

In the era of COVID-19, utilization of telemedicine has dramatically increased. Our QI initiative demonstrated high patient satisfaction along with time savings in a publicly funded health care system. Studies suggest that Clinical Immunology and Allergy may be uniquely situated to benefit from permanent integration of virtual care into regular practice for both new and follow-up appointments [1–3, 18]. We anticipate continued increased utilization of telemedicine, signifying a lasting beneficial change in the delivery of healthcare.

## Data Availability

The datasets used and/or analyzed during the current study are available from the corresponding author on reasonable request.

## Abbreviations

CIA: Clinical immunology and allergy
QI: Quality improvement

## References

[1] Thomas I, Siew LQC, Rutkowski K (2021). Synchronous telemedicine in allergy: lessons learned and transformation of care during the COID-19 pandemic. J Allergy Clin Immunol Pract.

[2] Shaker MS, Oppenheimer J, Grayson M, Stukus D, Hartog N, Hsieh EWY (2020). COVID-19: pandemic contingency planning for the allergy and immunology clinic. J Allergy Clin Immunol.

[3] Krishna MT, Knibb RC, Huissoon AP (2016). Is there a role for telemedicine in adult allergy services?. Clin Exp Allergy.

[4] Lee E, Song C, Vadas P, Morgan M, Betschel P (2021). Retrospective analysis of synchronous telemedicine use in Clinical Immunology and Allergy (CIA): a population-based cohort study in Ontario, Canada. J Allergy Clin Immunol.

[5] Waibel KH, Bickel RA, Brown T (2019). Outcomes from a regional synchronous tele-allergy service. J Allergy Clin Immunol Prac.

[6] Phadke NA, Wolfson AR, Mancini C, Fu X, Goldstein SA, Ngo J (2019). Electronic consultations in allergy/immunology. J Allergy Clin Immunol Pract.

[7] Mustafa SS, Yang L, Mortezavi M, Vadamalai K, Ramsey A (2020). Patient satisfaction with telemedicine encounters in an allergy and immunology practice during the coronavirus disease 2019 pandemic. Ann Allerg Asthma Immunol.

[8] Lanier K, Kuruvilla M, Shih J (2021). Patient satisfaction and utilization of telemedicine services in allergy: an institutional survey. J Allergy Clin Immunol Pract.

[9] Alvarez-Perea A, Sánchez-García S, Muñoz Cano R, Antolín-Amérigo D, Tsilochristou O, Stukus DR (2019). Impact of "eHealth" in allergic diseases and allergic patients. J Investig Allergol Clin Immunol.

[10] Shiff B, Frankel J, Oake J, Blachman-Braun R, Patel P (2021). Patient satisfaction with telemedicine appointments in an academic andrology-focused urology practice during the COVID-19 pandemic. Urology.

[11] Ontario Ministry of Health and Long Term Care. Resource Manual for Physicians. http://www.health.gov.on.ca/English/providers/pub/ohip/physmanual/download/section_4.pdf. 1 Sept 2021.

[12] Middlesex-London Health Unit. Summary of COVID-19 cases in Middlesex-London. https://www.healthunit.com/covid-19-cases-middlesex-london. 1 Sept 2021.

[13] Public Health Ontario. Ontario COVID-19 data tool: COVID-19 daily case counts and rates by reported date in Ontario. https://www.publichealthontario.ca/en/data-and-analysis/infectious-disease/covid-19-data-surveillance/covid-19-data-tool?tab=trends. 15 Jan 2022.

[14] Appireddy R, Khan S, Leaver C, Martin C, Jin A, Durafourt B (2019). Home virtual visits for outpatient follow-up stroke care: cross-sectional study. J Med Internet Res.

[15] Berlin A, Lovas M, Truong T, Melwani S, Liu J, Liu ZA (2021). Implementation and outcomes of virtual care across a tertiary cancer center during COVID-19. JAMA Oncol.

[16] Habashi P, Bouchard S, Nguyen GC (2019). Transforming access to specialist care for inflammatory bowel disease: the PACE telemedicine program. J Can Assoc Gastroenterol.

[17] Mustafa SS, Vadamalai K, Ramsey A (2021). Patient satisfaction with in-person, video, and telephone allergy/immunology evaluations during the COVID-19 pandemic. J Allergy Clin Immunol Pract.

[18] Zhu R, Kim H, Jeimy S (2020). Appointment characteristics during COVID-19 restrictions: a Canadian allergy/immunology centre perspective. J Allergy Clin Immunol Pract.

